# Functional circuits and signal processing in the enteric nervous system

**DOI:** 10.1007/s00018-020-03543-6

**Published:** 2020-05-18

**Authors:** Candice Fung, Pieter Vanden Berghe

**Affiliations:** Laboratory for Enteric NeuroScience (LENS), Translational Research Center for Gastrointestinal Disorders (TARGID), University of Leuven, Leuven, Belgium

**Keywords:** Enteric circuitry, Neurons, Glia, Microbiota, Neuroimmune, Epithelium

## Abstract

The enteric nervous system (ENS) is an extensive network comprising millions of neurons and glial cells contained within the wall of the gastrointestinal tract. The major functions of the ENS that have been most studied include the regulation of local gut motility, secretion, and blood flow. Other areas that have been gaining increased attention include its interaction with the immune system, with the gut microbiota and its involvement in the gut–brain axis, and neuro-epithelial interactions. Thus, the enteric circuitry plays a central role in intestinal homeostasis, and this becomes particularly evident when there are faults in its wiring such as in neurodevelopmental or neurodegenerative disorders. In this review, we first focus on the current knowledge on the cellular composition of enteric circuits. We then further discuss how enteric circuits detect and process external information, how these signals may be modulated by physiological and pathophysiological factors, and finally, how outputs are generated for integrated gut function.

## Introduction

The enteric nervous system (ENS) is critical for orchestrating gut motility, secretion, and blood flow along the entire gastrointestinal tract. These functions are in turn essential for a variety of gut processes including digestion and absorption, passage of content, as well as maintenance of body fluid homeostasis. The ENS also plays an important role in host defense, for instance by increasing motility and secretion to expel noxious agents, and via neuro-epithelial and neuroimmune interactions [1, 2]. The importance of this neural network is exemplified not only by the life-threatening consequences when enteric neurons fail to develop, such as in Hirschsprung disease [3–5], but also in more subtle conditions when there are faults in ENS wiring during development [6] and intestinal transit is severely delayed. Furthermore, neuronal degeneration in the ENS may also contribute to gastrointestinal symptoms commonly reported in neurodegenerative disorders such as Parkinson’s disease and Alzheimer’s disease [7]. For a clinically focused perspective, we direct readers to an excellent review by Schneider and colleagues [8], which comprehensively covers these many roles of the ENS in regulating gastrointestinal function and in the context of disease.

The ENS is an elaborate neural network equipped with a complete repertoire of intrinsic sensory neurons (intrinsic primary afferent neurons, IPANs), excitatory and inhibitory interneurons, and motor neurons, as well as enteric glia. These basic elements form the building blocks of the enteric circuits underlying integrated gut function, enabling the system to receive external inputs, integrate information, and generate outputs. The millions of enteric neurons and glia within the gut wall are arranged in two ganglionated and interconnected plexus layers: the myenteric and submucosal plexus [9]. The myenteric plexus, situated between circular and longitudinal muscle layers, is well positioned for its key role in coordinating gut movements. The submucosal plexus situated beneath the mucosal epithelium lining the lumen is mainly involved in the control of water and electrolyte secretion, and blood flow. Indeed, all functional elements within the intestine including the epithelium, smooth muscle, interstitial cells of Cajal (ICC), vasculature, and immune cells are innervated by the ENS, allowing the gut to monitor and exert appropriate responses to the dynamic external environment. While the ENS can operate autonomously, it also receives and integrates extrinsic inputs from the central nervous system (CNS) [10].

Although many fundamental principles have been elucidated, our understanding of various aspects of integrated ENS function remains limited owing to its complex organization, particularly with regards to its interactions with the microbiome and immune system [8]. With its components interspersed among a multitude of different cell types within the contractile intestine, studying the influence of luminal contents on functional enteric circuits has traditionally posed as a technically difficult task. Nevertheless, advances in imaging and genetic technologies have and will continue to facilitate this process [11]. This review highlights recent advances in unraveling the circuitry of the ENS and its diverse functions, the mechanisms by which local factors modulate its activity, and revisits some longstanding questions that remain to be addressed.

## Cellular components of the enteric circuitry

Early descriptions of peristaltic reflexes in the intestine operating independently of the CNS were provided by Von Haller in 1755 [12]. Later, in 1899, Bayliss and Starling presented evidence that this reflex activity is attributed to local intrinsic nerve circuits present in the gut [13]. Around the same time, Dogiel published illustrations of three morphological subtypes of neurons in the ENS, defined as Dogiel Types I, II, and III [14]. It was not until 1974 that Hirst and colleagues then reported on their electrophysiological properties and classified them into AH- and S-type neurons [15]. AH neurons are typified by their larger action potentials (APs) with an inflection on the falling phase and long afterhyperpolarizing potential (AHP; > 2 s) that follows. By comparison, S neurons are characterized by their brief APs that lack slow AHPs, and they display fast excitatory postsynaptic potentials (EPSPs). Subsequently, around the 80–90s, Costa, Furness, and colleagues conducted an extensive series of immunohistochemical and electron microscopy studies to group enteric neurons based on their distinct neurochemistry, projection patterns, and ultrastructure [16–29]. These were further correlated with their electrophysiological and functional properties [30–34]. Collectively, these morphological, electrophysiological, and neurochemical classification systems, informed by anatomical and functional studies, constitute the foundation of our standard enteric neuronal nomenclature and knowledge of the basic enteric circuitry.

Current studies often rely on inferring the function of neurons based on their neurochemical identity by immunostaining for the expression of primary transmitters or their synthesizing enzymes, and cytoskeletal and calcium-binding proteins. However, due to obvious technical considerations, i.e., the number of different antisera that can applied at once is restricted, this methodology severely limits the possibility to further identify heterogeneity within major functional subtypes of enteric neurons. Moreover, the neuronal markers applied may not necessarily directly correlate with their function. However, studies using various single-cell RNA-sequencing methods optimized for the ENS are now emerging, allowing for extensive gene expression profiling of functional molecular constituents of enteric neurons such as ion channels, transmitters and their synthesizing enzymes, as well as receptors [35, 36]. This methodology based on statistical clustering algorithms subdivided myenteric neurons into nine molecularly defined subpopulations. By comparison, ten different classes of myenteric neurons were identified from integrated anatomical, neurochemical, functional, and pharmacological analysis [10]. While this approach still provides indirect evidence for different functional subtypes, this information will undoubtedly generate important clues to facilitate our interpretation of the composition of enteric circuits and their higher order functions.

The most basic enteric circuit consists of a limited number of cellular elements: a sensory neuron directly, or via an interneuron, synapsing onto an excitatory or inhibitory output motor neuron. Although this minimal circuit is theoretically sufficient to elicit peristalsis, it has come to light that a fourth essential component, that is, the glial cell, also actively participates in modulating intestinal motility and secretion [37, 38], and possibly also in neuroimmune interactions [39]. These functional neuroglial units are distributed in an overlapping and repetitive fashion around the circumference and along the length of the gut. Further complexity arises with its convoluted organization, where heterogeneous populations of enteric neurons that innervate different targets are mixed within clusters of interconnected ganglia, making the decoding of this system exceptionally challenging.

### Intrinsic sensory neurons

Intrinsic sensory neurons (or IPANs) are typically polymodal, possessing chemosensory and mechanosensory properties, and have Dogiel Type II morphology [40]. Furthermore, they are associated with AH-type characteristics where their AHP enables them to set a level of excitability through somatic gating of activity within the network [41]. This in turn allows them to transduce the intensities, durations, and patterns of physiological stimuli. While intrinsic sensory neurons are often considered one population, single-cell sequencing data provides evidence for several subclasses [35, 36]. One clear division is whether they are situated in the submucosal or myenteric plexus. Only the former subset are sensitive to subtle mechanical stimulation of the mucosa, i.e., local mucosal deformation by pressure ejection from a micropipette [42, 43].

**Fig. 1:**
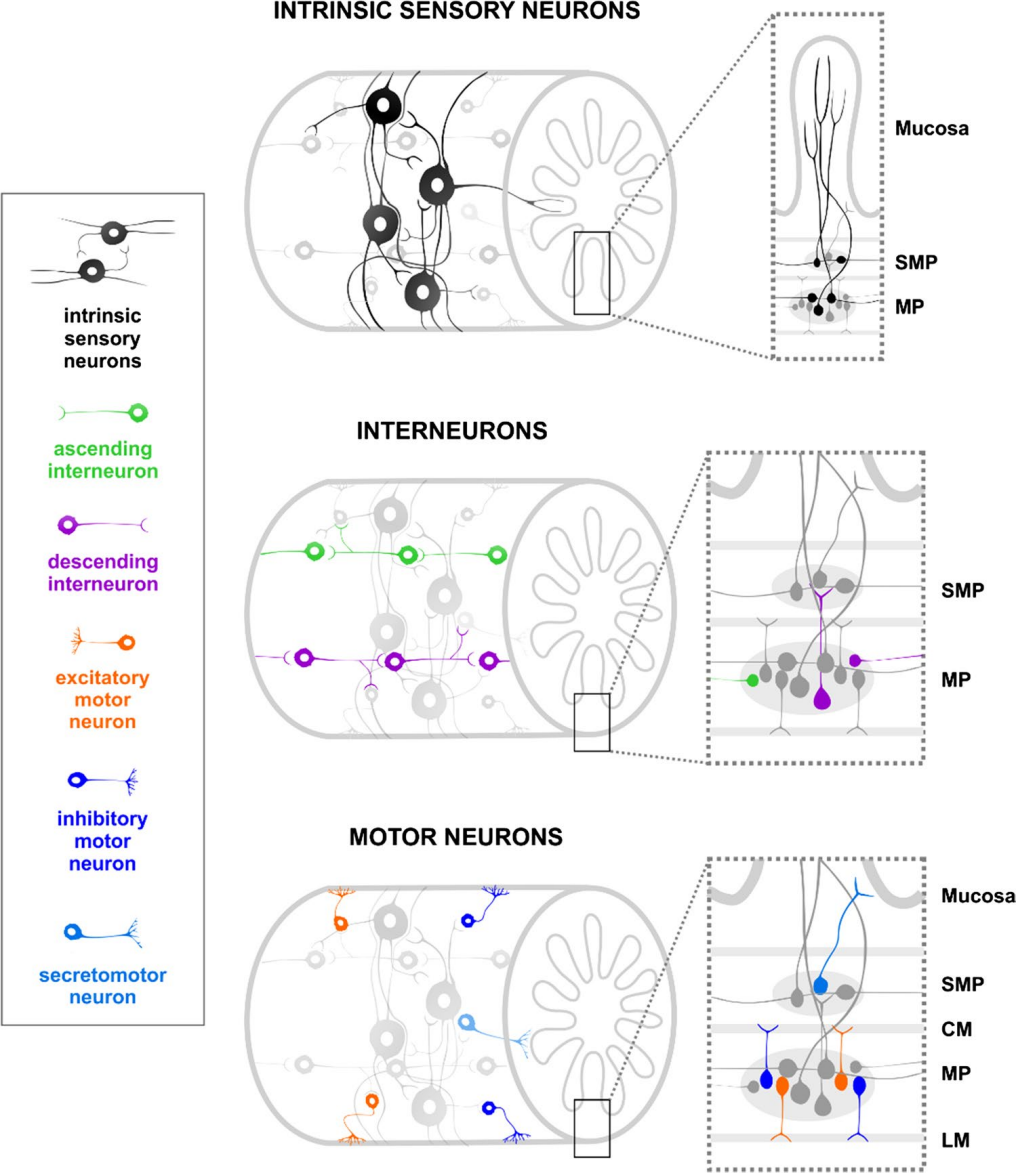
Neuronal components of the enteric circuitry. Intrinsic sensory neurons form interconnected networks encompassing the circumference of the gut wall, and provide extensive innervation of the mucosal epithelium. Within the myenteric plexus (MP), interneurons form chains along the length of the gut, with ascending interneurons projecting orally and descending interneurons projecting anally. Myenteric excitatory and inhibitory motor neurons innervate the circular (CM) and longitudinal muscle (LM), while secretomotor neurons in the submucosal plexus (SMP) project to the mucosa

Intrinsic sensory neurons predominantly project circumferentially almost encircling the full gut tube, but also extend local projections longitudinally in both directions, and provide extensive innervation of the mucosal epithelium [44] (Fig. 1). All their projections have transmitter release sites. These sensory neurons form interconnected networks which enable them to integrate and reinforce information not only locally but across a distributed network [41]. In this sense, intrinsic sensory neurons can act as interneurons. They also provide excitatory input to all other functional classes of enteric neurons [45].

In addition to Dogiel Type II (AH) neurons, a population of enteric neurons with Dogiel Type I morphology (identified as interneurons) can also behave as intrinsic mechanosensing neurons in the colon [46], and similarly in the small intestine, where mechanosensory properties were found in myenteric neurons that do not share morphological features with Dogiel Type II neurons [47].

### Interneurons

Additional to intrinsic sensory neurons that can also function as interneurons [46], there are uniaxonal myenteric neurons that project orally or anally, i.e., ascending and descending interneurons, respectively (Fig. 1). The different neurochemical classes of interneurons can differ between gut regions. For instance, in the guinea pig ileum, there is one class of excitatory ascending interneurons, and three classes of descending interneurons, while in the colon, there are three neurochemical classes of ascending interneurons, and four classes of descending interneurons [48, 49]. Such regional differences may contribute to the different motor patterns that they display. Neurons within each of these different subclasses can interconnect with like neurons to form chains extending along the length of intestine. Ascending interneurons also receive inputs from local sensory neurons and project to excitatory motor neurons, while descending interneurons receive inputs from local sensory neurons, and project to inhibitory motor neurons. Some interneurons may also provide inputs to sensory neurons. Furthermore, descending interneurons, but not ascending interneurons, project to submucosal ganglia. Acetylcholine (ACh) is generally the primary transmitter of interneurons, but each subtype may use various co-transmitters or neuromodulators such as 5-hydroxytryptamine (5-HT), adenosine triphosphate (ATP), tachykinin (TK), nitric oxide (NO), and somatostatin (SOM) [45].

### Motor neurons

In the myenteric plexus, excitatory and inhibitory motor neurons supply the circular and longitudinal muscle to evoke muscle contraction or relaxation (Fig. 1). Excitatory motor neurons tend to project orally and utilize ACh as their primary transmitter, while inhibitory neurons project anally and use various co-transmitters including NO, vasoactive intestinal peptide (VIP), and Pituitary adenylate cyclase-activating polypeptide (PACAP) [45]. In the submucosal plexus, secretomotor and vasodilator neurons innervate the mucosa and submucosal vasculature to regulate intestinal secretion and blood flow, respectively [50]. The two key transmitters of submucosal neurons are ACh and VIP [45, 51, 52].

### Enteric glia

Although enteric glia have previously been less extensively studied than their neuronal counterparts, it has become apparent that they actively participate in regulating various gut functions [53], including the regulation of colonic motility in physiology [37, 54–57] and in pathophysiology such as colitis [58–61].

Enteric glial subtypes (I–IV) have been described based on their distinct morphology and location within the layers of the gastrointestinal tract, as well as different response signatures [62]. As with enteric neurons, enteric glia also label differentially for various neurochemical markers such as Glial fibrillary acidic protein (GFAP), Sox10, and S100 calcium-binding protein β (S100β), but the various patterns of GFAP, S100β, and/or Sox10 are not unique to any one morphological glial subtype [62]. Through single-cell sequencing studies, seven distinct classes of enteric glial cells have been identified [35]. However, how their morphological features, spatial distribution, and genetic profile relate to functional diversity remains to be determined.

## Inputs to enteric circuits

### Intrinsic sensory pathways

The gut is constantly subject to a plethora of different sensory stimuli including chemical and mechanical signals present in the luminal contents, as well as mechanical feedback from the contracting muscle. The ENS must be able to detect this information, integrate it, and regulate its activity to generate appropriate outputs accordingly. Thus, intrinsic sensory circuits are essential for the gut to monitor this dynamic environment. Morphologically, intrinsic sensory neurons are multipolar and branch extensively, giving rise to two routes by which APs may propagate. The majority of APs cross the soma to reach other efferent processes, while some bypass the cell body via an axon branch (i.e., axon reflexes). Thus, the integration of sensory inputs may occur at the level of the nerve process, in the soma, or at the level of the network [41]. Much of the information processing and sensory signal integration that occurs within this population of sensory neurons is thought to largely determine the functional output of the intestine. This is most apparent in simple monosynaptic reflexes comprising an intrinsic sensory neuron and a motor neuron, where mucosal stimulation triggers a burst of fast EPSPs in inhibitory motor neurons and corresponding inhibitory junction potentials in the muscle that are similar in time course to the burst of APs recorded in the sensory neuron [41, 43]. However, the detection of mechanical stimuli in the gut may not be so straightforward. There is evidence that mechanosensitivity is not limited to the classic intrinsic sensory neuron, but is a property of a broader population of enteric neurons [63].

#### Acute detection of luminal nutrients

There is an extensive innervation of the mucosal epithelium from intrinsic sensory neurons, but these nerve endings do not come into direct contact with the luminal contents. Specialized enteroendocrine cells (EECs) scattered throughout the epithelium are responsible for first sensing the luminal milieu and then communicating these signals to the ENS. EECs are known to be widely activated by nutrient stimulation [64, 65]. Accordingly, they are equipped with an array of molecular machinery to sense different chemicals within the lumen as well as mechanical distortion of the mucosa [66–68]. EECs also produce a range of signaling peptides and hormones to further transduce the signal through paracrine or endocrine mechanisms [69]. While chemosensory transduction in the epithelium has been extensively examined, our knowledge of the specific molecules involved in EEC signaling to the ENS is surprisingly lacking. EECs make specialized contacts via their ‘neuropod’ with extrinsic sensory afferents innervating the epithelium [70–72], but the specificity of the intrinsic enteric circuitry involved in detecting different luminal stimuli remains unclear. The local application of various nutrient stimuli, and acidic or basic solutions to the mucosa can activate enteric neurons [43], but the exact mucosal mediators involved in the communication between EECs and enteric neurons remain elusive. Single-cell RNA-sequencing of small intestinal epithelium shows that EEC-subtype-enriched genes encode for key peptides [substance P, glucagon-like peptide 1 (GLP-1), cholecystokinin (CCK), neurotensin, secretin, glucose-dependent insulinotropic polypeptide (GIP), and ghrelin] as well as synthesizing enzymes for signaling amines (e.g. 5-HT) [69, 73]. Complementary to these data, single-cell RNA-sequencing of enteric sensory neurons indicate that they express genes encoding for many of the corresponding receptors including *HTR3a* (5-HT_3_ receptor), *Tacr1* (Neurokinin 1 (NK1) receptor), and *Glp1r* (GLP-1 receptor) [35, 36]. Perhaps, one of the most well-characterized signaling pathways of mucosal chemosensory transduction is 5-HT release from EC cells, which then activates 5-HT_3_ receptors on intrinsic sensory nerve endings in the mucosa to further transmit the luminal signal [67, 74–76]. Nonetheless, the specificity of neuro-epithelial circuits involved in detecting different luminal constituents and subsequent signaling within the ENS are still ill defined.

#### Long-term interactions with symbiotic organisms

The intestinal microbiota is a component of the luminal content that has gained much attention given its importance in gut homeostasis and implications in a diverse range of diseases including autism, obesity, and Parkinson’s disease [77]. The ENS is in a prime position to serve as a key interface through which microbiota can influence intestinal physiology [78]. Thus, how the microbiota signals to the ENS has been an area of considerable interest. Intrinsic sensory nerves may detect mucosal microbial products via various mechanisms including the chemosensation of bacterial metabolites via EECs, through microbiota-produced neurotransmitters such as 5-HT, dopamine, gamma aminobutyric acid (GABA), and ACh (although the concentrations of these produced in vivo is unknown), or via the activation of innate immune pathways by microbe-associated molecular patterns (MAMPs) signaling [1].

Short-chain fatty acids (SCFAs) are a major candidate for mediating crosstalk between microbiota and the ENS. Notably, the most prominent metabolites of gut microbial fermentation are the SCFAs’ acetate, propionate, and butyrate [79]. In the epithelium, many EECs express several G-protein-coupled receptors (GPCRs) through which SCFAs may act, including the fatty acid receptor 2 (FFAR2 or GPR43) and 3 (FFAR3 or GPR41). FFAR3 is also expressed by a subset of enteric neurons [80]. Whether SCFAs signal to neurons via signaling mediators released from EECs or may diffuse through the epithelium to directly act on underlying enteric neurons, or both, remains unclear. Nonetheless, commensal bacteria acting via SCFAs have been shown to increase the density of EC cells and upregulate* Tph1* expression (*Tph1* encodes the synthesizing enzyme for 5-HT), which, in turn, impacts ENS activity by modulating serotonergic signaling [81, 82].

Microbial factors may also act via various Toll-like receptors (TLRs) which specifically recognize the molecules of microbial origin, i.e. MAMPs. Lipopolysaccharide (LPS, the major membrane component of Gram-negative bacteria) via TLR activation stimulates hormone release and secretion of inflammatory mediators from mouse EEC cell lines [83]. However, the effect of these mediators on activity of enteric neurons is not well understood. As with receptors for SCFAs, the overlapping expression of TLRs on epithelial EECs (TLR1, 2, 4, 5, 6, and 9) [83, 84], and enteric neurons (TLR2, 3, 4, 7, and 9) [85–87], as well as glia [88] makes it challenging to identify the relative contributions of specific receptor pathways to ENS activation.

Although the specific enteric circuits through which microbiota may act and how they are modulated by microbes are not well defined, it is clear that microbial composition impacts the electrophysiological properties of myenteric intrinsic sensory neurons. Two studies by McVey Neufeld et al. [89, 90] showed that myenteric AH neuronal excitability was decreased in germ-free mice compared to specific pathogen-free control mice and conventionalized germ-free mice, as evidenced by a reduction in the number of APs generated at 2× threshold. The slow AHP was also reported to be prolonged in germ-free mice, although the underlying mechanism is unclear [89]. Nonetheless, it is apparent that the gut microbiota plays a key role in setting the excitability of enteric neurons and it is likely that this results in the summation of multiple microbial factors acting via various signaling pathways. Most recently, Obata et al. identified a novel molecular mechanism linking luminal microbiota to the regulation of colonic motility. They show that microbiota, via activation of acryl hydrocarbon receptor (AhR) signaling, determine the transcription profile of enteric neurons, and as a net result affect colonic motility [91].

### Extrinsic inputs

The ENS is often described to be capable of functioning autonomously in its local regulation of intestinal motility and secretion, independently of the CNS. Nonetheless, gastrointestinal function ultimately depends on the integration of both local enteric and central nervous activity. The ENS, depending on the gut region, receives various forms of efferent input from extrinsic neurons including vagal, spinal thoracolumbar, and spinal lumbosacral innervation [10, 92]. Vagal efferent motor neurons supplying the upper GI tract are cholinergic and originate from the nucleus ambiguous and the dorsal motor nucleus in the brain stem. These pre-enteric pathways are mainly responsible for regulating esophageal propulsion, gastric distension, and antral contractility. While the majority of gastric enteric neurons receive vagal innervation, there is only a relatively minor contribution of vagal efferent inputs to the distal intestine and colon, both structurally and functionally [10]. By contrast, enteric neurons of the small and large intestines are densely innervated by sympathetic efferent nerves, which have their cell bodies located in the prevertebral ganglia and utilize noradrenaline as their primary transmitter [10]. Enteric glia have also been found to receive sympathetic efferent input [93]. These sympathetic neurons act to slow intestinal transit and reduce intestinal secretion [10]. Towards the distal end of the gut, the pelvic innervation of the distal colon and rectum is responsible for maintaining fecal continence and coordinated emptying of the bowels via the ENS [10, 94].

## Modulation of the ENS

Collectively, the summation of all the inputs that the gut receives critically determines the level of activity within the enteric neuronal network and subsequent output that it generates. However, the properties of enteric circuits are also subject to modulation by various physiological and pathophysiological factors.

### Physiological modulators

#### Mechanosensory feedback

Mechanical feedback informs the gut of its physical state, which is essential for effective mixing, propulsion, or storage of the containing intestinal contents. Both mechanical distortion of the mucosa and muscle distension are detected by enteric neurons. While the classic model of GI neuronal mechanosensitivity attributes this property to IPANs, where distortion of their cell bodies or processes triggered action potential discharge [95, 96], there is increasing evidence that a broader population of enteric neurons, that is, including S-type neurons, also display mechanosensitive properties [46, 47, 63] and these have been termed more generally ‘mechanosensitive enteric neurons’ (MEN) [97]. Thus, it is possible that motor neurons have the potential to regulate their own output directly via feedback from the muscle. While it has been demonstrated that the detection of mucosal distortion is mediated via mechanosensitive EECs [67, 68, 98], it is unclear if there are mechanosensitive nerve endings that are also directly activated.

#### Neuroglia signaling

Enteric glial cells express a plethora of (predominantly G-protein-coupled) receptors for a broad range of neurotransmitters and modulators in the gut, and thus, they have the capacity to monitor and respond to enteric neurotransmission [99]. The most well-studied form of enteric neuron-to-glia communication is purinergic signaling and enteric glia express various nucleotide receptors including P2Y1 [100–102], P2Y4 [103–105], and A2B receptors [106, 107], as well as eNTPDase2, which hydrolyses ATP (released from enteric neurons) to ADP—a glial cell agonist [99]. Furthermore, structurally defined neuroglia units have been described in the ENS. A mechanism by which enteric neuronal cell bodies signal to a defined number of surrounding glia was identified, where neuron-to-glia communication occurs via purinergic signaling through pannexin channels [108]. There is increasing evidence from functional studies to suggest that activated enteric glia in turn modulate various gut functions and this will be discussed in the following sections.

#### Macrophages

Self-maintaining gut macrophages closely situated near blood vessels, submucosal and myenteric neurons and nerve fibers, Paneth cells, and Peyer’s patches have recently been described. These macrophages were found to exert niche-specific functions in intestinal homeostasis, including the regulation of intestinal secretion and motility [109]. In particular, muscularis macrophages confined to the muscularis externa have been shown to modulate peristalsis by a direct action on enteric neurons via the secretion of the growth factor bone morphogenetic protein 2 (BMP2), while enteric neurons reciprocally regulate macrophage numbers via colony stimulating factor (CSF1). Furthermore, this interaction between enteric neurons and muscularis macrophages can be influenced by signals from the microbiota which tunes the levels of BMP2 and CSF1 [110].

### ENS modulation in pathophysiology

Crosstalk between the enteric neurons, glia, and intestinal immune cells is important in inflammation. Their mode of communication is via the release of various mediators including cytokines, neurotransmitters, and neuromodulators. While neurons do not form synapses onto immune cells, their axons come into close proximity to immune cells and various inflammatory mediators can, in turn, act on the ENS [2].

#### Noxious agents and allergens

Whereas the lack of microbiota decreases AH neuronal excitability, a common characteristic of gut inflammation and infections—whether it be due to bacteria, viruses, parasites, toxins or dietary allergens—is that AH neurons become hyperexcitable with an attenuation of its AHP through slow EPSPs. Although, the specific mechanisms by which ionic currents are altered may differ and are not completely understood. cAMP is thought to be a key mediator of slow EPEPs, with the primary slow EPSP-evoking transmitter being the tachykinin, substance P. Indeed, cholera toxin from the diarrhoea-inducing bacterium *Vibrio cholera* appears to induce hyperexcitability in myenteric AH neurons via tachykinins and NK3 receptors [111]. The elevation in cAMP may also result from the release of other factors that act through adenylyl cyclase (AC), including inflammatory and immune mediators, e.g., histamine and prostaglandins. For instance, sustained AH cell hyperexcitability following infection with the GI parasite *Trichinella spiralis* was associated with upregulated AC cAMP signaling, protein kinase A (PKA) activation [112], and histamine release [113]. Similarly, in guinea pigs sensitized by milk ingestion, hyperexcitability in colonic submucosal AH neurons was associated with histamine release from mucosal mast cells acting on H_2_ receptors, which couple to AC [114]. While in the TNBS (2,4,6-trinitrobenzene sulfonic acid) model of inflammation, there is evidence for the involvement of prostaglandin E2 (PGE2), since Cox-2 inhibition prevents the AH neurons from becoming hyperexcitable [115].

#### The role of enteric glia in inflammation

Enteric glia actively participate in inflammatory states and can respond to various pro-inflammatory mediators, e.g., cytokines, and when activated can promote the secretion of proteins such as S100β, which subsequently triggers NO production by inducible NOS (iNOS) and increases oxidative stress to induce neuronal damage [116]. In colitis, NO production from glial iNOS induces ATP release via the opening of glial connexin-43 (Cx43) hemichannels [60]. ATP generated from activated glia can in turn stimulate a signaling complex comprising neuronal purinergic P2X7 receptors, pannexin-1 channels, the adaptor protein ASC, and caspases to mediate neuronal cell death [101]. It has also been shown that increased iNOS activation in enteric glia contributes to disrupted epithelial permeability in colitis and this was restored by perturbing enteric glial function using fluoroacetate [59]. However, contrary to its intended purpose, fluoroacetate itself may in fact induce reactive gliosis and only has a minor effect on glial Ca^2+^ signaling at the population level [117].

Another mechanism by which enteric glia may be activated during inflammation was recently shown to involve tachykinins [118]. Thus, tachykinins not only contribute to inflammation through their effect on neurons, but also via mechanisms involving neuron–glia signaling. Tachykinins acting through NK1 and NK2 receptors on enteric neurons were shown to recruit glia by stimulating ATP release and activating P2Y1 receptors. This then drives glial ATP release via Cx43 hemichannel opening to trigger P2X7-mediated inflammation.

#### Macrophages

In addition to their role in homeostasis, macrophages can also contribute to inflammation. Enteric neurons and vagal neurons interact with macrophages to form a cholinergic anti-inflammatory pathway [119]. Vagal nerve stimulation can cause ACh release from cholinergic myenteric neurons onto closely apposed α7nAChR-expressing muscularis macrophages. This in turn inhibits ATP-induced calcium signaling in the macrophages, which attenuates their activation and was seen to reduce inflammation in a murine model of postoperative ileus.

#### Mast cells

Mast cells are mainly found in the lamina propria and submucosal plexus, where they are closely associated with the intestinal nerves [120]. In rodent models, mast cells respond to a variety of neurotransmitters including substance P, calcitonin-gene related peptide (CGRP), ATP, and SOM [121]. Since mast cells are generally sparse under physiological conditions and are more abundant during inflammatory states, for instance with food allergies or parasite infections [113, 114], it is likely that mast cell to nerve signaling is more prominent in disease states. Accordingly, a Ca^2+^ imaging study of the human submucosal plexus showed that under physiological conditions, communication from mast cells to nerves was rarely observed when mast cells were activated by IgE receptor crosslinking, which induces the release of a cocktail of mediators including proteases, histamine, and cytokines [122, 123]. On the other hand, nerve to mast cell signaling could be readily triggered by electrical stimulation [122].

## Functional outputs of the ENS

### Intestinal motility

**Fig. 2:**
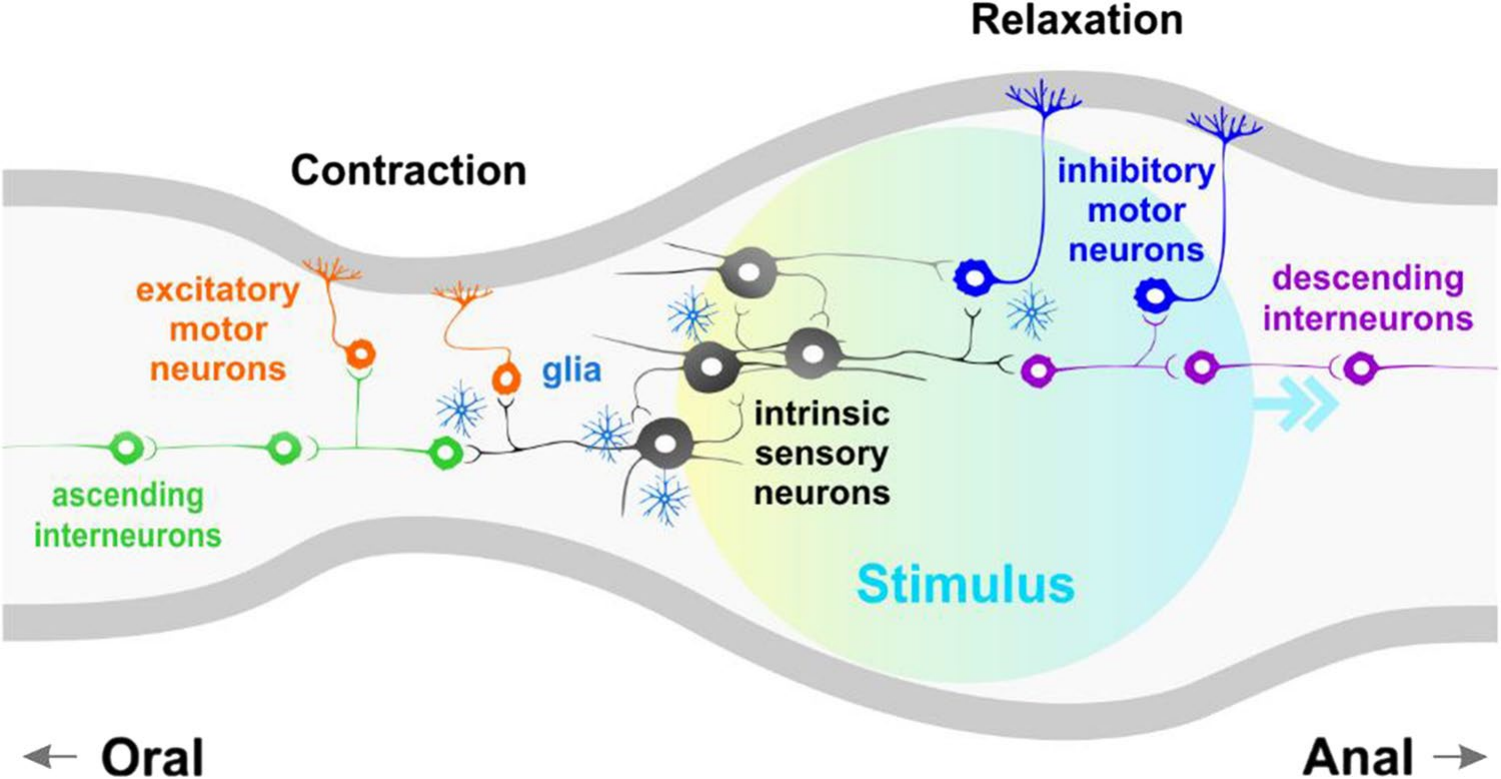
Simplified schematic representing the enteric circuitry underlying the peristaltic reflex. Intrinsic sensory neurons synapse with ascending and descending interneurons that form chains along the length of the intestine, as well as excitatory and inhibitory motor neurons. Interneurons also innervate motor neurons which in turn supply the circular and longitudinal muscle (musculature represented by grey lines; note that different muscle layers and their innervation are not defined). Upon detection of a luminal stimulus, the activation of ascending interneurons connected to excitatory motor neurons evoke a contraction orally, while the activation of descending interneurons connected to inhibitory motor neurons elicit a relaxation anally to propel the contents along. Enteric glia also plays an active role in regulating intestinal motility

Motility reflexes can be elicited by a broad range of chemical and mechanical stimuli including luminal nutrients, mechanical stimulation of the mucosa, and intestinal distension [124]. The GI tract displays a wide range of complex motor patterns to serve different purposes depending on the intestinal region and the nature of the sensory inputs received. Two of the major neurogenic contractile behaviors are segmentation contractions that mix luminal contents back and forth to optimize digestion and nutrient absorption [125–127], and peristalsis, a propagating contraction that propels content along the tract [13, 128–130]. The basis of most propulsive motility patterns is peristaltic reflexes which involve the activation of ascending interneurons which synapse excitatory motor neurons to evoke a contraction immediately oral to the stimulus site, while a concurrent activation of descending interneurons contacting motor neurons evokes relaxation anally [124] (Fig. 2).

While the underlying mechanisms of neurogenic peristalsis have been largely resolved [131], the circuits involved in other motility patterns such as mixing, and especially how the gut transitions between different motility patterns remain elusive. Since excitatory and inhibitory motor neurons are polarized, with ascending interneurons coupled to excitatory motor neurons and descending interneurons coupled to inhibitory motor neurons, monosynaptic reflexes would produce oral contractions and anal dilatations, not the stationary contractions seen in mixing motor behaviors. A combined computational modelling and in vitro study demonstrated that spatially localized contractions can arise from a local imbalance in ascending excitatory or descending inhibitory muscle inputs due to variations in the activity within ascending or descending reflex pathways [132]. It was proposed that variations in the activity within these pathways may be mediated by locally released neuromodulators, or may result from receptor desensitization or synaptic rundown. Furthermore, an observation from in vitro studies is that stationary contractions often occur repeatedly at the same location [127]. It was shown that this could be induced by an incision made through the myenteric plexus and muscle layers around the full gut circumference to severe ascending and descending pathways, where localized contractions occur either side of this lesion when exposed to a nutrient solution [132]. This suggests that structural differences may give rise to localized activity. Physiologically, this may be the number of neurons or synaptic contacts. Additionally, a prerequisite of this model was that the sensory input into the ascending and descending pathways is distributed along the length of the intestine to first leave the muscle in a resting state, which would then allow local variations to produce stationary contractions. Hence, a potential trigger for the switch from peristalsis to segmentation may be the shift from a localized to a distributed stimulus, or vice versa [132]. Another means of switching between propulsive and mixing patterns has been demonstrated by Ferens et al. who showed, using pharmacological agents, that this can be achieved by modulating the AHP of intrinsic sensory neurons [133].

Although intrinsic neural circuits are known to be important in mediating these various motor behaviors, how the gut transitions between states of quiescence and activity is another open question. Aspects of this have been recently studied in the context of rhythmic propulsive contractions which occur in the large intestine, termed colonic migrating motor complexes (CMMCs) [131]. Using mice that selectively expressed the Ca^2+^ indicator GCaMP3 in either cholinergic or nitrergic enteric neurons, it was shown that these two populations are differentially involved in coordinating CMMCs and tonic inhibition, respectively [134]. Further, optogenetic activation of channelrhodopsin2 (ChR2) in nitrergic neurons inhibited ongoing CMMCs. In a separate study, optogenetic activation of ChR2 in cholinergic calretinin neurons was shown to increase CMMCs and colonic propulsion and transit in vitro. It was further demonstrated using wireless optogenetics that colonic transit could also be increased in vivo, in conscious, freely moving mice [135]. Another study combined fluorescent Ca^2+^ imaging with electrophysiology to examine the activity of large populations of nitrergic inhibitory and cholinergic excitatory myenteric neurons simultaneously. It was found that both populations displayed a rhythmic firing pattern and discharge during CMMCs at the same time in repetitive bursts [136]. This supports the previous findings that showed disinhibition during CMMCs was not due to a “switching off” of a population of enteric neurons, but rather presynaptic inhibition of tonic inhibitory neurotransmitter release [137]. However, it is yet to be determined whether the behavior displayed by these neuronal populations arises from a specific set of pacemaker neurons, or emerges from the enteric network, such that it is not evident from examining the properties of individual neurons alone.

The diversity of intestinal motor behaviors exhibited can vary depending on the given gut region and the tasks required of it. For instance, the proximal colon exhibits a relatively diverse repertoire of motility patterns including anti-peristaltic waves that mix contents to maximally reabsorb water and electrolytes from the lumen, while the distal colon predominantly generates peristaltic contractions with its primary role in fecal pellet propulsion. Recent work suggests that this depends at least in part on the specific connectivity of the enteric circuitry in these different regions. Ca^2+^ imaging was performed at low magnification to study responses to focal electrical stimulation in a large population of neurons within a single field of view [138]. Data from this study indicate that there are more diverging, polysynaptic connections present in proximal colon as compared to the distal colon, which mainly utilizes monosynaptic circuits and is more dependent on nicotinic transmission. Indeed, recent electrophysiological studies also confirm that different neurogenic patterns of electrical activity exist in the smooth muscle of the proximal colon, compared with the distal colon [139].

#### Interstitial cells of Cajal (ICCs)

In the myenteric plexus, interstitial cells of Cajal (ICCs) (ICC-MY) serve as pacemakers and generate rhythmic patterns of depolarizing activity in the muscle termed slow waves [140]. It has been suggested that motor neurons innervate ICCs, and ICCs may in turn play a role in neurotransmission by transducing neuronal signals to regulate muscle activity [141]. For instance, an earlier study in the gastric fundus showed that excitatory junction potentials in the smooth muscle evoked by cholinergic excitatory inputs were attenuated in mutant mice lacking most intramuscular ICC [142]. However, in mutant mice lacking pacemaker-type ICC and electrical slow waves (at least in the small intestine), the neurogenic migrating motor complexes (MMCs) still occur and these mice have no apparent gastrointestinal problems [143]. Nonetheless, there is yet to be a consensus on the contribution of ICC to motor neurotransmission, and this topic has been extensively reviewed by others [144–146].

#### The role of enteric glia in intestinal motility

Fluorescent Ca^2+^ imaging studies have demonstrated that activity within the enteric glial network appears to be synchronized with nerve activity during CMMCs [55] and this glial activity is important for intestinal motility [57, 102]. Interestingly, enteric glia have also been reported to regulate GI motility in a sex-specific manner [37], where the ablation of PLP-1 expressing glia decreased GI transit time and increased CMMC frequency in female, but not male mice. While the involvement of enteric glia in the regulation of intestinal motility is becoming apparent, still little is known regarding the specific transmitter pathways involved. Although enteric glia in the myenteric plexus express both M3 and M5 muscarinic receptors, it has been shown that muscarine-evoked Ca^2+^ responses in glia are mainly mediated by M3 receptors [147]. The functional role of glial M3 receptor signaling was further examined using a DREADDs (designer receptors exclusively activated by designer drugs) system, where mice were generated to express a modified human M3R (hM3Dq) exclusively on GFAP^+^ glia that can be directly and selectively activated by the synthetic ligand clozapine-N-oxide (CNO). CNO activation of enteric glia enhanced colonic motility, suggesting an endogenous role of glial M3 receptor signaling in modulating intestinal motility [147]. In addition to ACh, other neurotransmitters including 5-HT and ATP, which mediate fast synaptic neurotransmission, also stimulate enteric glia in vitro [148]. However, whether these endogenous transmitters signaling pathways are involved in enteric glial modulation of motility reflexes, and the mechanism by which enteric glia may exert effects on intestinal motility still requires further study.

### Intestinal vasodilation and secretion

The coupling of intestinal secretion and vasodilation is crucial for the movement of water and electrolytes across the mucosal epithelium [149]. These processes are largely regulated by vasomotor and secretomotor neurons located in the submucosal plexus. Depending on the species, these may be mutually exclusive populations or the same neurons which innervate both the epithelium and vasculature. The two major subtypes of vasodilator/secretomotor neurons are often distinguished by their expression of VIP or choline acetyltransferase (ChAT) [50, 52, 150, 151].

#### Vasodilator reflexes

**Fig. 3:**
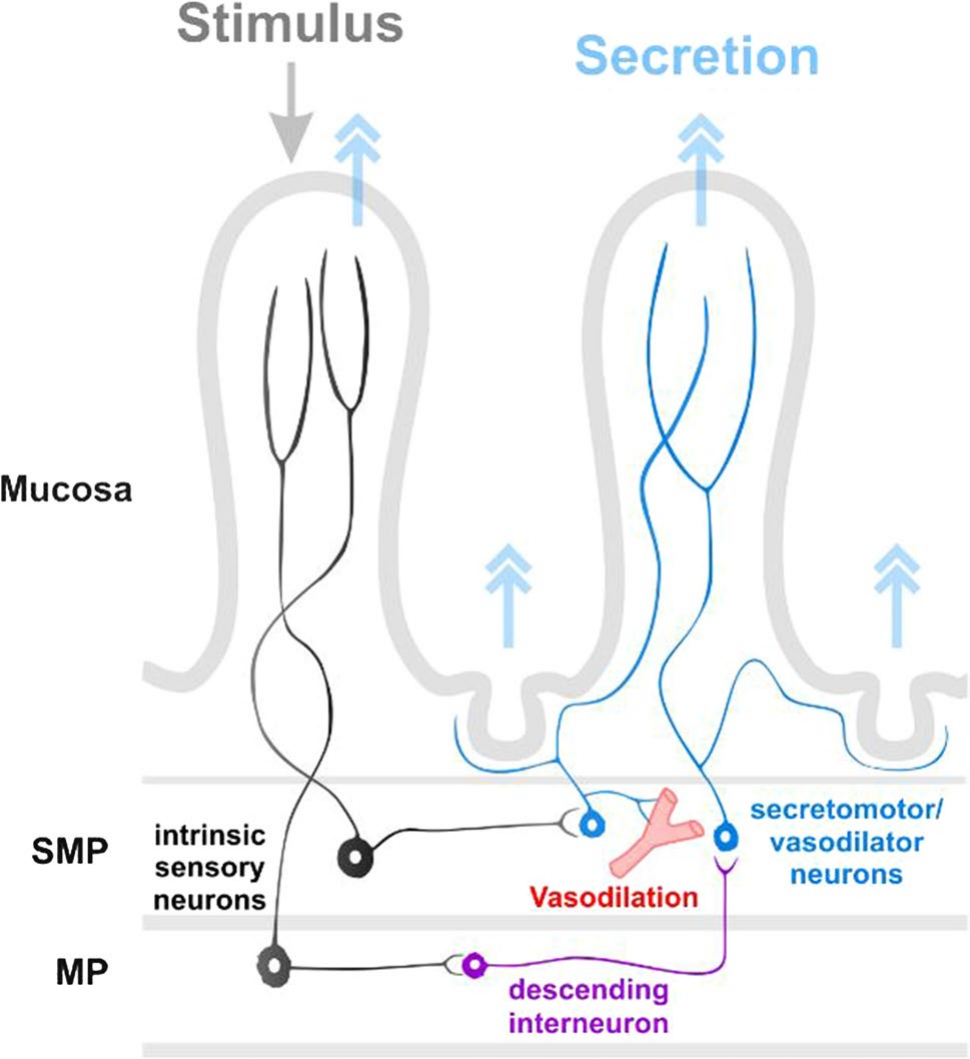
Schematic illustrating neurons involved in vasodilator and secretomotor reflexes. Chemical or mechanical stimulation of the mucosa activates myenteric and/or submucosal sensory neurons which then excite secretomotor/vasodilator neurons directly or via interneurons to stimulate secretion and/or vasodilation. Intrinsic sensory neurons may also directly evoke a secretory response via an axon reflex

Intestinal blood flow is critical for maintaining intestinal epithelial health, supplying the water and electrolytes to the secretory epithelium, and for fueling its metabolic needs, especially during digestion [8, 149]. Studies of vasomotor reflexes have demonstrated that the enteric innervation of the vasculature is responsible for vasodilation and is important in the local physiological regulation of mucosal blood flow, while vasoconstriction regulated by the extrinsic nerves supplying the gut has been proposed to have a more prominent role in inflammatory states [152]. Local vasodilator reflexes can be triggered by chemical or mechanical stimulation of the mucosa. Studies in guinea pig small intestine have also provided evidence for the presence of short vasodilator reflexes confined to the submucosal plexus and long vasodilator reflexes that run through the myenteric plexus [50, 152, 153] (Fig. 3). The activation of these intrinsic pathways leads to the release of ACh onto endothelial cells to activate muscarinic M3 receptors, which triggers NO release and evokes vasodilation [152]. In human studies, postprandial hyperemia has been shown to involve cholinergic neurons, although it is unclear whether they were of intrinsic or extrinsic origin [154].

#### Secretomotor reflexes

Distension [155], mechanical distortion of the mucosa [156, 157], luminal chemicals and nutrients [158], and various noxious agents [159] all trigger secretomotor reflexes (Fig. 3). The submucosal plexus may contain both the efferent and afferent elements of the secretomotor circuit, depending on the region and species. For instance, complete secretomotor circuits have been described in the submucosal plexus of guinea pig and rat colon [156, 157, 160]. These in vitro secretion studies using preparations with the myenteric plexus removed showed that mucosal stroking excites submucosal intrinsic sensory neurons and stimulates the release of endogenous purines to activate postsynaptic P2Y receptors on cholinergic and VIPergic secretomotor neurons. Subsequent ACh or VIP release activates mucosal muscarinic M3 receptors or VPAC1 receptors, respectively, to trigger a secretory response [161]. In addition to this conventional circuit, the activation of axon reflexes may cause substance P release from intrinsic sensory neurons with collaterals in the mucosa to directly activate epithelial NK1 receptors and stimulate secretion [162]. Retrograde tracing of submucosal neurons innervating the mucosa of guinea pig colon showed that the majority of their projections were less than 5 mm longitudinally or circumferentially [163]. This is consistent with the proposal that intrinsic secretomotor reflexes are predominantly localized and confined to the site of the stimulus, i.e., are short reflexes. However, there is also evidence for long secretomotor reflex pathways that run through the myenteric plexus in the guinea pig small intestine [164]. This study showed that applying an electrical stimulus to intact mucosa evoked fast EPSPs in submucosal neurons located 0.7–1 cm anally from the point of stimulation. The number of neurons displaying fast EPSPs was reduced by severing the connections between the submucosa and the underlying muscle and myenteric plexus layers. Furthermore, fast EPSPs were absent after a lesion in the myenteric plexus was made between the stimulus site and the recording site but were still observed if the lesion was made in the submucosal plexus. Thus, the reflex examined likely involves myenteric sensory afferents innervating the mucosa, and that the circuit activated runs through the myenteric plexus.

#### The role of enteric glia in intestinal secretion

Like the regulation of intestinal motility, enteric glia may also contribute to the control of intestinal secretion. Although the gliotoxin fluorocitrate (a metabolite of fluoroacetate) did not induce any apparent changes to intestinal secretion [165], it has been reported that glial-specific Cx43 knockout mice (in which the propagation of glial Ca^2+^ signals is disrupted) produce fecal pellets with higher water content [102]. Furthermore, the disruption of glial activity in Cx43 knockout mice attenuated neurogenic secretory responses [38] and selective glial activation can evoke secretory responses equal in magnitude to that elicited by neuronal depolarization. These responses were substantially reduced, but not abolished by the Na^+^ channel blocker tetrodotoxin (TTX). This suggests either the involvement of neurons expressing TTX-resistant Na^+^ channels, or that enteric glial may also modulate intestinal secretion via a mechanism that does not involve neurotransmission within the enteric circuitry, i.e., by directly signaling to epithelial cells.

### ENS contribution to epithelial homeostasis

Intestinal stem cells are important for the continual replenishment of the epithelium as it is replaced every 3–5 days [8, 166]. Intestinal stem cells are housed at the base of the crypts, a compartment of the epithelium that is less accessible to luminal contents and thought to rely more on neural regulation [167]. Epithelial composition critically depends on the regulation of intrinsic developmental signaling pathways in actively cycling Lgr5^+^ intestinal stem cells, which is in turn influenced by extrinsic environmental cues [168, 169]. These regulatory mechanisms must be tightly controlled to ensure sufficient proliferation for epithelial replacement, but must be limited to prevent hyperproliferation and tumor growth.

There is accumulating evidence for a modulating role of the ENS in epithelial proliferation where the involvement of various neuromediators (including ACh and 5-HT) have been implicated [8]. Notably, it has been suggested that ACh stimulates intestinal stem cell proliferation [170, 171]. The activation of 5-HT2A receptors on cholinergic neurons via neuronal 5-HT has also been associated with enhanced ACh release and epithelial proliferation [172].

Studies further point to an ENS contribution to the regulation of intestinal stem cell fate and differentiation [170, 173, 174]. Specifically, co-culturing murine small intestinal stem cells with enteric neurons and glia have suggested that the ENS contributes to promoting their differentiation into EECs [173]. It has also been demonstrated that vagal neural crest cells innervated pluripotent stem cell-derived human intestinal organoids when grown together in culture. Here, differentiated β-tubulin- and S100β-expressing neuronal and glial cells respectively influenced epithelial differentiation by regulating the expression of genes related to various epithelial phenotypes [175, 176].

### The innervation of Peyer’s patches and host defense

Peyer’s patches are a component of the gut-associated lymphoid tissue that is responsible for maintaining tolerance to food antigens and commensal microbes, as well as triggering immune responses to infection by luminal pathogens, such as *Salmonella* and *Shigella* [177]. The specialized follicle-associated epithelium covering the Peyer’s patches comprise ‘microfold’ M cells that capture antigens from the lumen and transport them into the underlying lymphoid follicular dome regions for processing by antigen-presenting cells [178]. Although M cells constitute a first line of defense, they can also be exploited by pathogens as a route of invasion [179]. The innervation of Peyer’s patches has been anatomically characterized by various studies and they are found to be more densely innervated by intrinsic and extrinsic fibers than other regions of the mucosa [180–182]. Extrinsic TRPV1^+^ dorsal root ganglia nociceptor neurons have been shown to play a critical role in host protection against *Salmonella typhimurium* [183]. However, the contribution of the intrinsic nerve supply to Peyer’s patches in host defense is less clear. The enteric innervation mainly originates from submucosal neurons including intrinsic sensory neurons [182]. It is likely that these submucosal neurons are involved in detecting invading pathogens and in regulating the follicular blood supply and lymphocyte trafficking [180–182]. It is also feasible that the activation of submucosal neurons and secretomotor reflexes triggers a hypersecretory response as a means of clearing the invading pathogen such as in cholera, salmonella, and rotavirus infections [159, 184, 185]. Notably, interleukin-8 derived from enteric neurons has recently been shown to regulate the production of antimicrobial protein by goblet cells, which target bacterial pathogens such as *S. typhimurium* [186].

## Conclusion and future perspectives

Much of the groundwork has been laid for understanding the various components of the enteric circuitry, but we are only beginning to comprehend how these elements are interconnected and interact to ultimately give rise to coordinated intestinal function. One substantial obstacle has been the technical limitations associated with simultaneously measuring the rapid changes in nerve activity and in motility and secretion. Moreover, earlier methodologies lacked the temporal and spatial resolution necessary to identify the specific neural elements involved. However, recent advances in imaging technology together with transgenic reporter mice have greatly facilitated the study of enteric network activity and the corresponding output underlying complex gut behaviors [187–191]. For example, synchronous activity of myenteric and submucosal neurons during CMMCs can be observed using fluorescent Ca^2+^ imaging [192]. While detailed analysis of the enteric circuitry still proves challenging in such studies with movement artefacts inherently being a limiting factor [188], analytical methods are being developed to address these issues [189]. On the other hand, the measurement of these intestinal movements and their correlation with nerve activity may provide an informative readout. Advances in intravital abdominal imaging techniques [193–195] also open up further possibilities for the addressing longstanding questions on the coordination of intestinal secretion and motility. With these developments, we are well positioned in our pursuit of a more comprehensive understanding of integrated gut physiology and we anticipate interesting times ahead.
